# Causality Analysis to the Abnormal Subcortical–Cortical Connections in Idiopathic-Generalized Epilepsy

**DOI:** 10.3389/fnins.2022.925968

**Published:** 2022-06-30

**Authors:** Yun Qin, Sipei Li, Dezhong Yao, Cheng Luo

**Affiliations:** ^1^Sichuan Provincial People’s Hospital, MOE Key Lab for Neuroinformation, School of Life Science and Technology, University of Electronic Science and Technology of China, Chengdu, China; ^2^Sichuan Institute for Brain Science and Brain-Inspired Intelligence, Chengdu, China; ^3^Glasgow College, University of Electronic Science and Technology of China, Chengdu, China

**Keywords:** idiopathic generalized epilepsy, dynamic functional networks, causal relationship, subcortical-cortical circuit, modulation

## Abstract

Idiopathic generalized epilepsy (IGE) was characterized by 3–6 Hz generalized spike-wave discharges (GSWDs), and extensive altered interactions in subcortical-cortical circuit. However, the dynamics and the causal relationship among these interactions were less studied. Using resting-state functional magnetic resonance imaging (fMRI) data, the abnormal connections in the subcortical-cortical pathway in IGE were examined. Then, we proposed a novel method of granger causal analysis based on the dynamic functional connectivity, and the predictive effects among these abnormal connections were calculated. The results showed that the thalamus, and precuneus were key regions representing abnormal functional network connectivity (FNC) in the subcortical-cortical circuit. Moreover, the connectivity between precuneus and adjacent regions had a causal effect on the widespread dysfunction of the thalamocortical circuit. In addition, the connection between the striatum and thalamus indicated the modulation role on the cortical connection in epilepsy. These results described the causality of the widespread abnormality of the subcortical-cortical circuit in IGE in terms of the dynamics of functional connections, which provided additional evidence for understanding the potential modulation pattern of the abnormal epileptic pathway.

## Introduction

Idiopathic generalized epilepsy (IGE) was typically characterized by 3–6 Hz generalized spike-wave discharges (GSWDs). The mainstream concept believed that the burst of GSWD was related to the inter-regional interactions in the subcortical-cortical circuit (Blumenfeld, 2003, 2005), involving the abnormal connectivity between the thalamus and cortex, and also the altered connectivity among the prefrontal and sensorimotor area (Szaflarski et al., 2010; Qin et al., 2019). Invasive animal studies have found that there was a large-scale burst of rhythmic oscillations in the thalamus during epileptic discharge (Avanzini et al., 2000; Timofeev and Steriade, 2004). Moreover, it was found that GSWDs were time-locked with BOLD activation of the thalamus and cortical deactivation, especially in the frontal cortex (Salek-Haddadi et al., 2003). In addition, even in the resting-state without GSWD, extensive abnormal functional networks and connections between networks were found in IGE, such as the default mode network (DMN), basal ganglia, and sensorimotor network (SMN) (Luo et al., 2011, 2012; Jiang et al., 2020; Parsons et al., 2020). However, the causal relationship among these abnormal connections was less studied, which may provide evidence for understanding the modulation mechanism within the epileptic pathway.

Regarding the origin of generalized epileptic discharge, previous studies have proposed some different discharge model hypotheses, with two representative concepts of the cortical focus theory and the thalamic pacemaker theory. The hypothesis of cortical focus theory assumed that epileptic discharges originated in a certain local area of the cortex and then spread through the interaction of subcortical structures with the cortex, thus recruiting specific neuronal networks into typical oscillatory behavior (Meeren et al., 2005; Stefan and Lopes da Silva, 2013). Under this concept, a general comment about the thalamus burst, was that a sustained flow of GSWD signals propagated from cortex to thalamus in a couple of hundreds of milliseconds, and triggered the oscillation entrainment in the cortical-thalamus-cortical loop. On other hand, the hypothesis of thalamic pacemaker suggested the reticular thalamic nucleus contained the pacemaker cells for the thalamic clock, imposing its rhythm to the cortex, then resulting to the spread of discharge activity in the cortex (Buzsaki, 1991; Avanzini et al., 1992). Moreover, studies have shown that interference with thalamus activity through stimulation can promote the termination of epileptic discharge (Berenyi et al., 2012; Paz et al., 2013; Burdette et al., 2020), indicating that the thalamus not only played an important role in epileptic seizures and transmission, but also modulated the termination of epileptic discharge. Besides the animal and model analysis, we think investigating the predictive relationship among the abnormal connections based on the dynamic functioning in the resting-state in thalamocortical circuit can provide additional evidence for understanding the origin and propagation the epileptic activity.

Other structures under the cortex, such as the striatum and cerebellum, also played an important role in regulating epilepsy. Some animal studies have shown that the striatum, whose fibers connected to the substantia nigra reticulum, was involved in the regulation of epileptic discharges (Turski et al., 1989; Deransart et al., 2000). Previous EEG–functional magnetic resonance imaging (fMRI) studies have shown that the putamen and caudate nucleus was closely related to epileptic discharges, and that the increase of epileptic discharges was accompanied by the enhancement of the internal connectivity within the basal ganglia network (BGN) (Luo et al., 2012; Moeller et al., 2013; Bartolini et al., 2014). It was generally assumed that the regulation of the GSWD of epilepsy by the striatum was achieved through its contribution to the thalamocortical circuit. On the other hand, the cerebellum was considered to be a potential regulator of epileptic discharge activity. It has been found that direct stimulation of the cerebellar cortex could effectively destroy the thalamus-cortex oscillations and thereby inhibit the activity of GSWD (Berenyi et al., 2012). Therefore, probing how these subcortical regions, i.e., striatum and cerebellum interacted with the epileptic circuit would be helpful for the modulation strategy of the potential clinical intervene.

In this study, we investigated the altered subcortical-cortical pathway of IGE, and the causality relationship among these abnormal connections was examined using the dynamic functional of the resting-state network. The dynamic interactions within the abnormal epileptic networks may provide evidence for the origin and modulation of epileptic activity.

## Materials and Methods

### Participants

In total, seventy-eight patients with IGE (40 women; mean age: 23.8 years) including 32 patients with juvenile myoclonic epilepsy (JME) and 46 patients with generalized tonic–clonic seizures (GTCSs) were recruited in this study. Diagnosis and classification was made by neurologists in accordance with the International League Against Epilepsy (ILAE) guidelines (Fisher et al., 2017; Scheffer et al., 2017). Routine CT and MRI examinations were conducted and no structural abnormality was found in all the epilepsy patients. Clinical information was showed in Table 1. In total, 60 healthy controls (29 women; mean age: 25.7 years) with no history of psychiatric or neurologic disorders participated in the study. Written informed consent according to the Declaration of Helsinki was obtained from all the participants. This study was approved by the Ethics Committee of the University of Electronic Science and Technology of China (UESTC).

**TABLE 1:** Summary demographic of patients and healthy controls.

	HC		Patients		χ^2^ t-test	
	*n*=60		*n*=78		*p value*	
Gender (Male/Female)	31/29		38/40		0.12	
Seizure type (JME/GTCS)	−		32/46		−	

	**Mean**	**Std**	**Mean**	**Std**		

Age(year)	23.8	2.6	25.7	9.6	0.474	
Seizure duration	−	−	6.75	6.45	−	−
Age at seizure onset	−	−	18.95	10.4	−	−

### Magnetic Resonance Imaging Acquisition

In this study, the MRI data of all the participants were recorded from the 3T MRI scanner (Discovery MR750, GE) with an eight channel-phased array head coil in UESTC. Resting-state fMRI data were acquired using a gradient-echo echo planar imaging sequences (FOV = 24 cm × 24 cm, FA = 90°, TR / TE = 2,000 ms/30 ms, matrix = 64 × 64, slice thickness/gap = 4 mm/0.4 mm). A total of 255 volumes were collected in the resting-state scan for each participant. During fMRI scanning, all the participants were instructed to keep still and close their eyes without sleeping. In addition, high-resolution T1-weighted images were also acquired using a 3D fast spoiled gradient echo (T1-3D FSPGR) sequence (FOV = 25.6 cm × 25.6 cm, FA = 9°, matrix = 256 × 256, TR / TE = 5.936 ms/1.956 ms, slice thickness = 1 mm, no gap, 152 slices).

### Functional Magnetic Resonance Imaging Data Pre-processing

fMRI data were pre-processed using SPM12^1^ and NIT toolboxes^2^ (Dong et al., 2018). The first five volumes were discarded from all fMRI scans for the magnetization equilibrium. The remaining volumes were slice-timing corrected using the first slice as reference, and spatially realigned to the mean of volumes within subject to correct head motion. We checked the head motion parameters after processing, the head motion of all participants was less than 2 mm translation and less than 2°rotation in any direction. Individual T1 images were co-registered to the functional images, and segmented into gray matter, white matter, and cerebrospinal fluid, and then normalized to the Montreal Neurologic Institute (MNI) space. Then, the functional images were spatial normalized based on T1 transformation matrix, resampled to 3 mm × 3 mm × 3 mm voxels, and spatially smoothed using a 6 mm full-width half maximum (FWHM) Gaussian kernel. Nuisance signals (12 motion parameters, linear drift signal, and also mean white matter and cerebrospinal fluid signals) were regressed out for the fMRI data to reduce the effect of potential artifacts.

### Causality Analysis to the Subcortical–Cortical Pathways in Idiopathic Generalized Epilepsy

**FIGURE 1:**
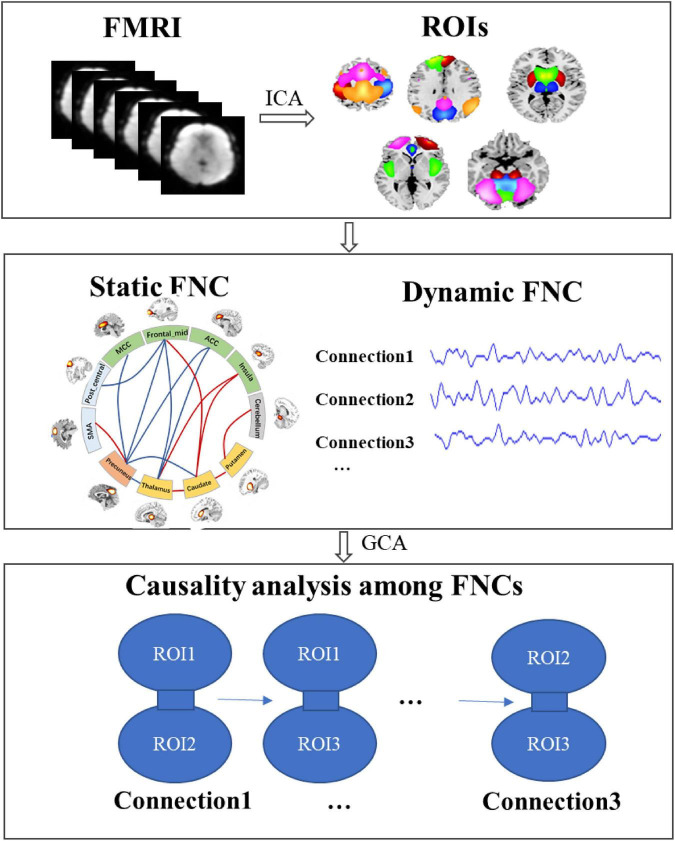
The diagram of this study to the detect the causality in subcortical–cortical pathways in IGE. First, the regions of interest (ROIs) were extracted using ICA, and then we constructed the static and dynamic functional network connectivity (FNC) among ROIs; furthermore, according to the abnormal static FNC in IGE and their dynamic courses across the slide time windows, granger causality analysis (GCA) was performed to examine the causal relationship among these abnormal connections.

This study aimed to investigate the alteration of subcortical–cortical pathways in IGE and the causality among these alterations. The main diagrams of this study was showed in Figure 1, including the following parts: (1) the regions of interest (ROIs) were extracted using ICA to the fMRI data, with the independent spatial patterns and the temporal courses being obtained; (2) we constructed the static and dynamic functional network connectivity (FNC) among ROIs; (3) according to the abnormal static FNC in IGE and their dynamic courses across the slide time windows, granger causality analysis (GCA) was performed to examine the causal relationship among these abnormal connections.

#### Extraction of Regions of Interest in Subcortical–Cortical Pathways

Spatial ICA was performed to the group fMRI, and the functional networks identified by the ICs were obtained. Here, the number of ICs was 100 and determined according to the previous studies. In ICASSO,^3^ the infomax algorithm was repeated 30 times to estimate the ICs. Then, dual regression approach was used in the back reconstruction to get the individual spatial maps and time courses. In this study, we focused on the subcortical and some frontal regions according to the previous studies, therefore, we selected the components of DMN, SMN, BGN, the salience network (SN), and also cerebellum network, each of network consisting of a few components, that is the ROIs. The MNI coordinates for the selected ROIs were shown in Supplementary Table 1. Then, the temporal coursed was band-pass filtered (0.01–0.1 Hz).

#### Static and Dynamic Functional Network Connectivity Analysis

The static FNC and dynamic FNC was constructed among ROIs. Static FNC was computed by the Pearson’s correlation of the time courses between any two selected components. Based on the static FNC, we calculated the degree of FNC nodes by summarizing the weights of the connections of each node. Dynamic FNC was also computed using correlations between windowed time-courses of different ROIs. Here, the length of the slide window was 50TR, with the sliding step width 1TR. The selection of the window length was constrained by the minimum frequency of the BOLD signal (f_*min*_ = 0.01 Hz). Then, the variation of the weights for the dynamic FNC in the time course was calculated, representing the dynamics of the connections in the subcortical–cortical circuit.

#### Causality Analysis of Functional Network Connectivitys

To detect the abnormal connections in IGE, two-sample *t*-test of the static FNC was performed. Based on the dynamic FNC weights, GCA was conducted to examine the causal relationship among these abnormal connections. Here, we used the residual-based bivariate GCA, and the main calculations were as follows:

First, autoregression models were constructed for two time series *X* and *Y*, which were the dynamic FNC weights of two abnormal connections in IGE.


(1) Xt=∑k=1pbk⁢X(t-k)+εt⁢Yt=∑k=1pb′k⁢Y(t-k)+εt′

Here, ε_*t*_ and $\varepsilon_{t}^{\prime }$ were the residual of the autoregressive model, *p* was the order of model. The variance of the two models can be expressed as:


(2) R⁢1=v⁢a⁢r⁢(εt)   T⁢1=v⁢a⁢r⁢(ε′t)

Then, the regression models of *X* and *Y* were combined, that is:


(3) $$X_{t}=\sum_{k=1}^{p}A_{k}\,X_{\left(t-k\right)}+\sum_{k=1}^{p}B_{k}\,Y_{\left(t-k\right)}+u_{t}$$

Yt=∑k=1pA′k⁢Y(t-k)+∑k=1pB′k⁢X(t-k)+u′t

Here, *u*_*t*_ and $u_{t}^{\prime }$ is the residual of the combined regression model, and their variance can be expressed as:


(4) R⁢2=v⁢a⁢r⁢(ut)   T⁢2=v⁢a⁢r⁢(u′t)

The influence of *X* on *Y* can be described as $F_{x\to y}=l\,n\,\frac{R\,1}{R\,2}$ and $F_{y\to x}=l\,n\,\frac{T\,1}{T\,2}$. Therefore, when the past values of *X* has a predictive effect on the current value of *Y*, *F*_*x→y*_ will be significantly larger than zero, that is, the directed connection from *X* to *Y*. Considering the non-normality of the distribution of *F*_*x→y*_, we used the transformed $F_{x\to y}^{\prime }$, as the alternative $F_{x\to y}^{\prime }$ and $F_{y\to x}^{\prime }$ representing an approximate normal distribution according to the previous studies, and the order *p* here was selected as 1 (Zang et al., 2012).


(5) F′x→y=[(t-p)⁢Fx→y-(p-1)3]1/2

F′y→x=[(t-p)⁢Fy→x-(p-1)3]1/2

Finally, $F_{x\to y}^{\prime }$ and $F_{y\to x}^{\prime }$ was *z*-scored at the individual level and then statistically analyzed using one-sample *t*-test (statistical threshold *P* < 0.01).

## Results

### Alteration of Functional Network Connectivity in Idiopathic Generalized Epilepsy

**FIGURE 2:**
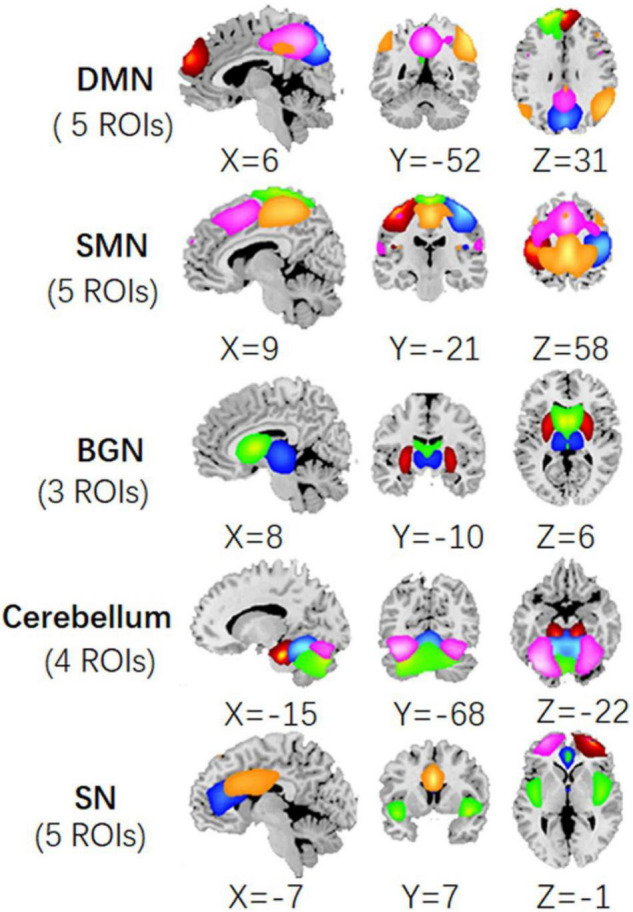
The ROIs extracted from the ICA processing of fMRI data, including precuneus, angular, PCC, frontal_sup_medial areas in DMN; bilateral post_central, pre_central, paracentral_lobule, and SMA in SMN; thalamus, putamen, and caudate in BGN; frontal_mid, ACC, MCC, and insula in SN; and cerebellum regions.

According to the previous studies, we selected the components in DMN, SMN, BGN, SN, and cerebellum from 100 ICs obtained in ICA analysis. Thus, each network was constituted of a few components, i.e., the ROIs. Here, DMN included the frontal_sup_medial area, the angular, cingulum_post (PCC), and two precuneus components. SMN included bilateral post-central areas, pre-central, paracentral_lobule, and supp_motor_area (SMA). BGN was comprised of the thalamus, putamen, and caudate. SN involved the left and right frontal_mid area, the cingulum_ant (ACC), cingulum_mid (MCC), and insula. The cerebellum network included the cerebellum posterior lobe and the declive. The maps of these five networks were illustrated in Figure 2. The central coordinates of the ROIs were shown in Supplementary Table 1.

**FIGURE 3:**
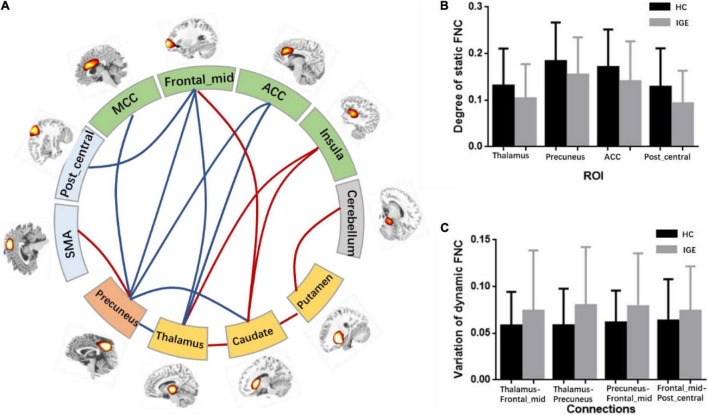
**(A)** Abnormal FNCs in IGE. This figure contained regions with significant alteration of FNC. The red lines represented the enhancement of connections in IGE and the blue lines represented the decrease of FNCs in IGE. **(B)** The degree of the static FNC nodes in IGE and HC, and significant decreased degree of thalamus, precuneus, ACC and post_central area was found in IGE. **(C)** The variation of dynamic FNCs in IGE and HC, and increased variation was found in thalamocortical and cortical–cortical circuit.

After the ROIs of the five networks were selected, the static FNC and dynamic FNC was constructed among ROIs. Then, two-sample *t*-test was conducted to the static FNC, and the difference of FNC between IGE and HC was shown in Figure 3 (*P* < 0.001). In between-group comparisons, age and gender were regressed out as the covariates. The results showed that thalamus, precuneus were the key nodes representing significant alteration in the subcortical–cortical circuit in epilepsy. Increased connectivity between thalamus and striatum, as well as the insula, and increased connectivity between precuneus and motor area was found in IGE comparing to HC. Decreased connectivity between the thalamus and frontal area, between precuneus and the frontal area, and also the thalamus/striatum was found in IGE. In addition, striatum showed increased connectivity with insula and cerebellum in epilepsy brain. Moreover, we compared the degree of FNC nodes between IGE and HC, and the thalamus, precuneus, ACC and post-central areas had decreased static FNC in IGE (*P* < 0.05) (Figure 3B).

Besides, comparison of static FNC was also performed between two epilepsy groups, i.e., JME and GTCS. Comparing to GTCS, JME showed decreased connectivity in thalamus-precuneus, and thalamus-frontal connections. The results may be related to the longer epilepsy duration of these patients with JME in this study.

### Causality Among the Abnormal Functional Network Connectivitys in Idiopathic Generalized Epilepsy

**FIGURE 4:**
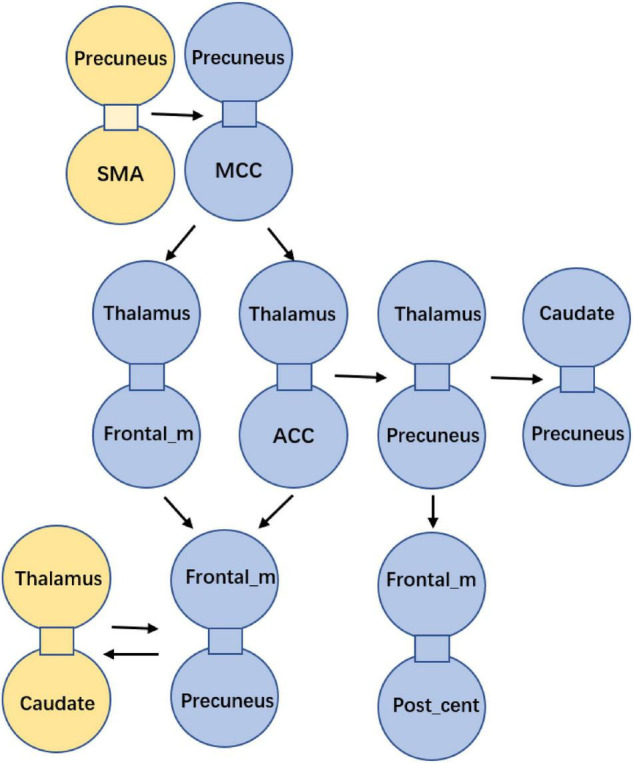
Causality among the abnormal FNCs in IGE. Each bounded sub-graph in the figure is one abnormal connection in IGE, with the yellow sub-graph denoting the increased connection, and the blue sub-graph denoting the decreased connection in IGE. The arrows denoted the predictive directions among the connections.

After the abnormal connections of static FNC in the epilepsy group were labeled, the dynamic changes of these connections were extracted according to the weights of the dynamic FNC matrices. The variation of the dynamic FNC showed that the connections of thalamus-frontal_mid, thalamus-precuneus, precuneus-frontal_mid, and frontal_mid-post_central had increased dynamics in IGE (*P* < 0.05) (Figure 3C). Then, the weights of the dynamic FNC were taken into the GCA processing. The directed GCA connections with statistical significance at the group level (*P* < 0.01) were shown in Figure 4. Hierarchical causal relationship was demonstrated between the connections. The connections between precuneus and nearby cortex, i.e., SMA and MCC, had predictive effects on the widespread connections between thalamus and frontal regions. Moreover, these decreased connections between thalamus and multiple cortexes also had predictive effects on the connectivity between frontal and other cortex, such as the precuneus and post-central area. The increased connectivity between thalamus and caudate had bidirectional causal relationship with the decreased frontal-precuneus connection.

**FIGURE 5:**
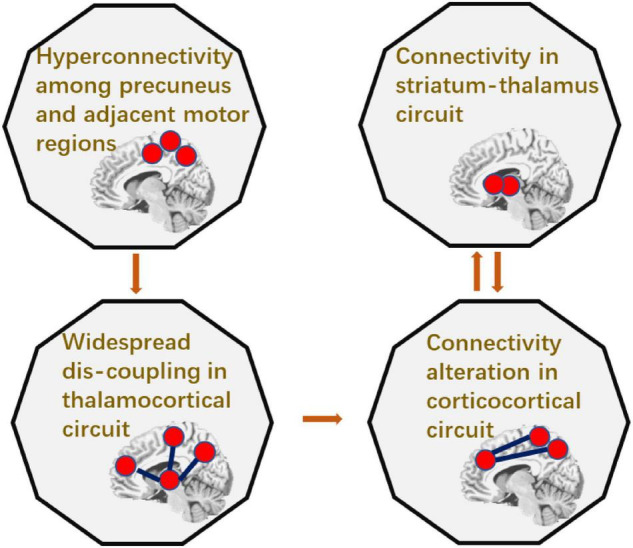
The summarization of the causality in epileptic circuit.

To sum up, patients with epilepsy had increased cerebellum-striatal-thalamic connections and widespread decrease in thalamocortical circuits. The causal relationship between these abnormal connections was summarized in Figure 5. Particularly, connectivity between precuneus and adjacent regions can predict the widespread dis-coupling in thalamocortical circuit. Moreover, the coupling between thalamus and the striatum had a two-way causal effect on the inter-cortical connectivity.

## Discussion

This study investigated the subcortical–cortical pathway in IGE, and showed that thalamus, precuneus were key nodes representing abnormal functional connectivity in subcortical–cortical circuit. Furthermore, the causality relationship among these abnormal connections in IGE was examined. It was shown that the increased connections between precuneus and the adjacent regions had the causal effect on the widespread decreased connections in thalamocortical circuit. In addition, the two-way causal effect of the connection between the thalamus and the striatum on the cortical connection indicated the modulation role of subcortical circuit in epilepsy.

Numerous studies have shown that there were abnormalities in a wide range of functional connections in IGE (Moeller et al., 2008; Vollmar et al., 2011; Jiang et al., 2018). Altered interactions in the subcortical–cortical and corticocortical circuit were thought to play an important role in the origin and propagation of epileptic activity (Luo et al., 2011; Benuzzi et al., 2012; Conradsen et al., 2013). The present study provided further evidence that the thalamus and precuneus were the key nodes of the abnormal subcortical–cortical circuit in IGE. A lot of decreased connections were found between thalamus and the cortical areas, involving the frontal and precuneus area. Meanwhile the connectivity between the frontal and precuneus area was also impaired. Moreover, the node degree of FNCs gave further evidence that thalamus and precuneus were important areas showing decreased connectivity in epilepsy. Furthermore, in the temporal dimension, the time-varying dynamics of the connections in thalamus-frontal_mid, thalamus-precuneus, precuneus-frontal_mid, and also the frontal_mid-post_central were significantly increased in IGE. The spatial dysconnectivity and the altered FNC dynamics in thalamocortical circuit in epileptic brain have been reported in many studies (O’Muircheartaigh et al., 2012; Kim et al., 2014; Zhang et al., 2018; Qin et al., 2020b). Previous studies have demonstrated the role of thalamus in leading in initiating and maintaining the epileptic activity, which may be involved early or late in the discharge (Tyvaert et al., 2009; Martin-Lopez et al., 2017). In addition, the striatum-thalamic-cortical circuit was considered to be the key circuit in the modulation of epileptic discharges (Paz et al., 2013). In the current study, intensive connectivity was found within BGN network, as well as between BGN nuclei and other brain regions, which implied the basal ganglia providing an endogenous control of thalamocortical SWDs.

According to the hypothesis of generalized epileptic discharges (Meeren et al., 2005), the epileptic discharge may originate from the focal area and the propagation involved abnormal cortical and subcortical interactions. In this study, the predictive relationship among these abnormal connections in epileptic brain was investigated using the mode-free granger causal analysis, which did not depend on the assumptions about the directions of the processes. The connections between precuneus and nearby cortex, i.e., SMA and MCC, had predictive effects on the widespread connections between thalamus and frontal regions. The precuneus has been reported to be involved in epileptic discharge network, with distinct activity changing within seconds before the discharge onset (Vaudano et al., 2009; Bai et al., 2010). Causal analysis showed that the precuneus gated the discharge activity in thalamocortical network, and was the region with the most occurrence of initial spikes and slow waves comparing to the thalamus and frontal area (Lee et al., 2014). Also, precuneus has been shown the strongest connectivity strength before epileptic discharges, and the increased connectivity between precuneus and the nearby cortex was found before discharges (Qin et al., 2020a). Therefore, this study demonstrated the contribution of the connectivity between precuneus and the motor-related area to the widespread dysfunction of thalamocortical circuit, which implied that the hyperexcitability and hyperconnectivity of precuneus may be one important trigger of epileptic discharges, thus, resulting to the wide dysconnectivity in thalamocortical circuit and the following altered connectivity between cortical areas.

In one of our previous study, we detected the synchronous network during the stage of epileptic discharges, and found the connectivity within frontal areas and between frontal–parietal areas was the main network pattern after the discharge onset (Qin et al., 2020a). Also, extensive signal enhancement in bilateral frontal regions was found both at discharge initiation and after discharge in IGE (Bai et al., 2010). In this study, the frontal areas were directly involved in the dysconnectivity of thalamocortical circuit and corticocortical interaction, which indicated the role of thalamus and frontal areas in propagation of epileptic discharges. In addition, it was interesting that the increased connectivity between thalamus and caudate had bidirectional causal effect on the decreased frontal–precuneus connection. Previous studies have shown that the circuit of striatum-thalamus modulated the cortical interactions in epileptic brain (Deransart et al., 2000). It was suggested that the modulation of BGN may be associated with the enhanced integration within BGN regions, i.e., the connectivity between striatum and thalamus, and this modulation effect on the inter-cortical connectivity may uncover the potential intervene mechanism of the deep brain stimulation in epileptic brain.

To re-test the stability of the causal analysis, we also conducted the multivariate GCA to describe the directed causal effect from this seed connection to the other connections, as well as the reverse impact intensities from other connections to this seed connection. The results showed similar directed patterns with the bivariate GCA, including the predictive effect from precuneus-related connections to the thalamocortical connections, and then to the cortical–cortical connections. Moreover, much extensive effects were found from the striatum-thalamus to both the thalamocortical and the cortical–cortical connections. The results were shown in Supplementary Figure 1.

To sum up the results, we concluded the causal relationship among the abnormal functional connectivity in epileptic brain, which described the trigger role of precuneus with its extensive hyperconnectivity, and the widely distributed dysconnectivity of thalamocortical circuit, and also the following alteration of frontal and motor-related connectivity. This causality in epileptic circuit provided additional evidence for the theory of epileptic origin and propagation on the level of macro functional connectivity. However, the main limitation of this study is that the causal relationship of the abnormal connectivity was based on the resting-state data rather than the discharging state. Therefore, the result provided the potential modulation pattern in terms of the epileptic activity, and the inference of the epileptic propagation circuit in this study was urgently needed to test by the physical stimulation and clinical evaluation.

## Conclusion

This study investigated the subcortical–cortical pathway in IGE, and showed that thalamus, precuneus were key regions of the abnormal FNC in epileptic brain. Furthermore, the causality analysis among these abnormal connections demonstrated the predictive effect of precuneus on the widely distributed thalamocortical dysconnectivity. In addition, the increased connectivity between thalamus and the striatum indicated the modulation role of subcortical circuit on the cortical connection in epilepsy. These results provided additional evidence for the widespread abnormality in epilepsy brain based on the dynamics of functional connections, and the predictive relationship in the subcortical–cortical circuit enlightened the theory of epileptic origin and propagation.

## Data Availability Statement

The raw data supporting the conclusions of this article will be made available by the authors, without undue reservation.

## Ethics Statement

The studies involving human participants were reviewed and approved by the Ethics Committee of the University of Electronic Science and Technology of China. The patients/participants provided their written informed consent to participate in this study.

## Author Contributions

YQ, DY, and CL conceived and designed the work. YQ and SL acquired the data. YQ analyzed the data. YQ and CL wrote the manuscript. All authors revised the work for important intellectual content and read, and approved the manuscript.

## Conflict of Interest

The authors declare that the research was conducted in the absence of any commercial or financial relationships that could be construed as a potential conflict of interest.

## Publisher’s Note

All claims expressed in this article are solely those of the authors and do not necessarily represent those of their affiliated organizations, or those of the publisher, the editors and the reviewers. Any product that may be evaluated in this article, or claim that may be made by its manufacturer, is not guaranteed or endorsed by the publisher.

## Footnotes

1
http://www.fil.ion.ucl.ac.uk/spm/

2
http://www.neuro.uestc.edu.cn/NIT.html

3
http://research.ics.tkk.fi/ica/icasso
