# Supplementary material for: Causality Analysis to the Abnormal Subcortical–Cortical Connections in Idiopathic-Generalized Epilepsy
Source: Front Neurosci. 2022 Jun 30;16:925968. doi: 10.3389/fnins.2022.925968 (PMC9280354; doi:10.3389/fnins.2022.925968)
Supplement: Supplementary file 1 [file Data_Sheet_1.docx]

**Supplementary Material**

**sFigure 1**


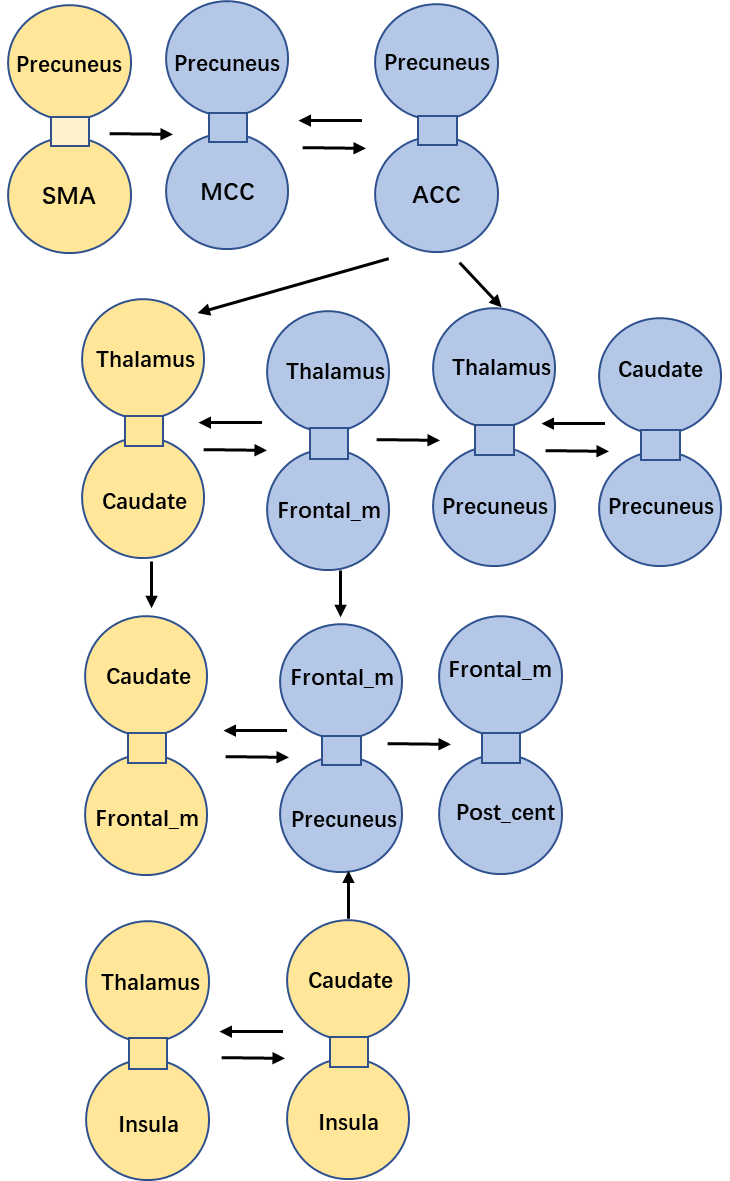


sFigure1. Causality relation among the abnormal FNCs in IGE using multivariate GCA. Each bounded sub-graph in the figure is one abnormal connection in IGE, with the yellow sub-graph denoting the increased connection, and the blue sub-graph denoting the decreased connection in IGE. The arrows denoted the predictive directions among the connections.

**sTable 1. The MNI coordinates of the selected ROIs.**
